# In Vitro Evolution of Allergy Vaccine Candidates, with Maintained Structure, but Reduced B Cell and T Cell Activation Capacity

**DOI:** 10.1371/journal.pone.0024558

**Published:** 2011-09-13

**Authors:** Ola B. Nilsson, Justus Adedoyin, Claudio Rhyner, Theresa Neimert-Andersson, Jeanette Grundström, Kurt D. Berndt, Reto Crameri, Hans Grönlund

**Affiliations:** 1 Department of Medicine, Karolinska Institutet, Stockholm, Sweden; 2 Swiss Institute of Allergy and Asthma Research (SIAF), University of Zürich, Davos, Switzerland; 3 Department Biosciences and Nutrition, Karolinska Institutet, Stockholm, Sweden; 4 Center for Allergy Research, Karolinska Institutet, Stockholm, Sweden; University of Cape Town, South Africa

## Abstract

Allergy and asthma to cat (*Felis domesticus*) affects about 10% of the population in affluent countries. Immediate allergic symptoms are primarily mediated via IgE antibodies binding to B cell epitopes, whereas late phase inflammatory reactions are mediated via activated T cell recognition of allergen-specific T cell epitopes. Allergen-specific immunotherapy relieves symptoms and is the only treatment inducing a long-lasting protection by induction of protective immune responses. The aim of this study was to produce an allergy vaccine designed with the combined features of attenuated T cell activation, reduced anaphylactic properties, retained molecular integrity and induction of efficient IgE blocking IgG antibodies for safer and efficacious treatment of patients with allergy and asthma to cat. The template gene coding for rFel d 1 was used to introduce random mutations, which was subsequently expressed in large phage libraries. Despite accumulated mutations by up to 7 rounds of iterative error-prone PCR and biopanning, surface topology and structure was essentially maintained using IgE-antibodies from cat allergic patients for phage enrichment. Four candidates were isolated, displaying similar or lower IgE binding, reduced anaphylactic activity as measured by their capacity to induce basophil degranulation and, importantly, a significantly lower T cell reactivity in lymphoproliferative assays compared to the original rFel d 1. In addition, all mutants showed ability to induce blocking antibodies in immunized mice.The approach presented here provides a straightforward procedure to generate a novel type of allergy vaccines for safer and efficacious treatment of allergic patients.

## Introduction

The domestic cat (*Felis domesticus*) is one of the most frequent pets and approximately 10% of the general population in industrialized countries is sensitized to cat allergens [1]. The symptoms deriving from cat allergy manifest mainly as rhinoconjunctivitis with a strong tendency to progress to asthma, especially in children [2].

Exposure to cat allergen is a result of the natural behavior of the cat to lick and groom. Proteins of the saliva is left to dry and spread as small airborne particles [3] where it can be detected in a variety of indoor environments [4], [5]. The major allergen of the cat, formally termed Fel d 1, is a hetero-dimer member of the secretoglobin protein family [6], [7] found in skin, lachrymal glands and in particular in the saliva. More than 95% of cat allergic patients show elevated serum IgE levels to Fel d 1 which is, therefore, the primary target for the development of immunotherapeutic vaccines for the treatment of cat allergy [8], [9].

Allergen-specific IgE is the key molecule for the development of allergic symptoms. The synthesis of IgE requires a B cell to undergo class switch recombination in close contact with allergen-specific T helper 2 cells (Th2) [10].

The T cell receptor on primed CD4+ T cells recognize antigen presenting cells carrying MHC II molecules with tightly bound enzyme-digested linear polypeptides of 12 to 25 amino acids derived from the primary structure of the antigen [11]. B cells expressing allergen-specific IgE as a part of the B cell receptor (BCR) recognize the surface structures of the allergens. After allergen binding, the receptor-antigen complex is internalized, the antigen processed, and displayed as peptide-MHC II complex on the B cell surface [12]. These processes link the 3D structure of the allergen to the linear peptides presented to the T cell receptor by the MHC class II complex.

Allergen-activated T cells are responsible for late-phase reactions (LPR), occurring 6–8 hours after allergen challenge eliciting symptoms ranging from oedema and itching to eczema, and also play a prominent and pivotal role in the pathogenesis of allergic asthma [13] as shown by the elevated numbers of CD4^+^ T cells found in bronchial mucosa, BAL fluid, and sputum in these patients.

The most common treatment of allergic diseases is either alleviation of symptoms using drugs or advice the patient to avoid the allergen source [14], [15]. However, allergen-specific immunotherapy (SIT), is the only treatment able to cure allergic diseases [16]. Successful SIT is thought to act through tolerance mechanisms induced by regulatory T cells and blocking IgG antibodies [17]. Induction of IgG antibodies may reduce clinical symptoms in several ways, by competition with IgE for binding epitopes on the allergens [18], [19], by inhibition of IgE-facilitated antigen presentation to CD4^+^ T cells [20], but also by interference with mast cell degranulation by down-regulation of IgE receptors signaling via inhibitory motifs on the FcγRIIb receptor [21]. Numerous studies have shown that crude allergen extracts currently used in SIT are clinically effective [22], a high allergen dose is more effective [23], although the potential risk of severe acute side effects is a limiting factor [24]. Attenuated allergenic molecules, i.e. hypoallergens or synthetic peptide fragments have been used as high dose and safer alternatives to conventional extract-based SIT [25], [26]. However, such treatments have also been limited by recurring side effects, such as LPR [27], [28].

T cell epitope (TCE) mappings of Fel d 1 have shown a scattered distribution of TCE located on both chains of Fel d 1 [29]. The relevance of T cells specific for Fel d 1 in cat allergic patients has been evaluated, where short overlapping peptides covering the major TCE of Fel d 1 elicited adverse effects, such as allergic rhinitis and late asthmatic reactions [28], [30], despite the fact that the peptides were unable to bind IgE, and thus elicit immediate allergic reactions. Interestingly, immunotherapy using selected allergen-specific Fel d 1 peptides, which induced tolerance to the allergen in a part of the T cell population, induced also suppression of T cells specific for epitopes which were not present in the peptide mixture used for treatment [31]. The use of recombinant hypoallergens containing the full spectrum of TCE has been reported in one multicenter clinical study [32]. The study included hypoallergens with an approximately 1000 fold reduced IgE bindning capacity, allowing a maintenance dose of 80 ug of allergen per injection. The large majority of the local and systemic reactions occurred several hours after injection [27].

Due to the close interactions between allergen, T, B and basophil or mast cell effector cells, the optimal allergy vaccine should be depleted of IgE-binding epitopes to evade cross-linking of high affinity receptors on effector cells and IgE-facilitated allergen presentation, and show a moderate T cell activation profile to avoid LPR and bronchial hypersensitivity. Modern biotechnology allows fast, easy cloning and production of allergens, allergen isoforms and hypoallergen variants with reduced IgE binding but conserved T cell reactivity [17], [33].

In this study we used a novel strategy to engineer full sized and folded allergy vaccine candidates with reduced number of T cell epitopes and reduced risk of inducing anaphylaxis, while maintaining the immunogenic properties. The design has several advantages over previously established methods for construction of hypoallergenic derivatives, which mainly involve disruption of structural B-cell epitopes, while essentially preserving T-cell reactivity. Although treatment with hypoallergens may be efficacious, it also carry the risk of inducing LPR [27], [28]. The rational of the strategy is based on selection of IgE-binding allergens from randomly mutated phage-displayed libraries of the major cat allergen Fel d 1, used for proof of concept. Because the vast majority of the solved protein-antibody complexes show discontinuous epitopes encompassing more than one surface loop [34], allergens selected by this technology are expected to recognize molecules conserving the natural conformation [35]. Therefore it should be possible, by consecutive error-prone PCR (epPCR) amplification of the primary Fel d 1 gene sequence, to construct mutated phage display libraries containing proteins with a maximal number of mutations, still allowing a native like fold of the allergen. Using this novel procedure, we have selected four candidates with reduced number of IgE binding epitopes and reduced T cell stimulating capacity. Recombinant proteins were produced, purified and evaluated for solubility, purity and native-like folding. T cell activity was assessed using cultured peripheral blood mononuclear cells (PBMC) from cat allergic patients, IgE binding by inhibition ELISA, and allergenicity via basophil activation test. The ability of the hypo-TCE candidate vaccines to induce IgE-blocking IgG-antibody responses *in vivo* was shown by mouse immunization experiments.

## Results

### Library construction and panning

Following epPCR, ligation and transformation, the generated mutated Fel d 1 phage libraries typically contained 10^5^ individual clones.

**Figure 1:**
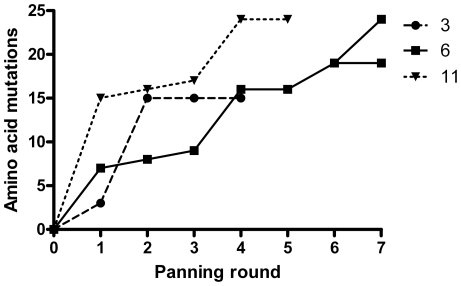
*In vitro* evolution of mutations by phage display. Number of amino acid exchanges (y-axis) in the four Fel d 1 mutants displayed as a function of panning rounds (x-axis).

**Figure 2:**
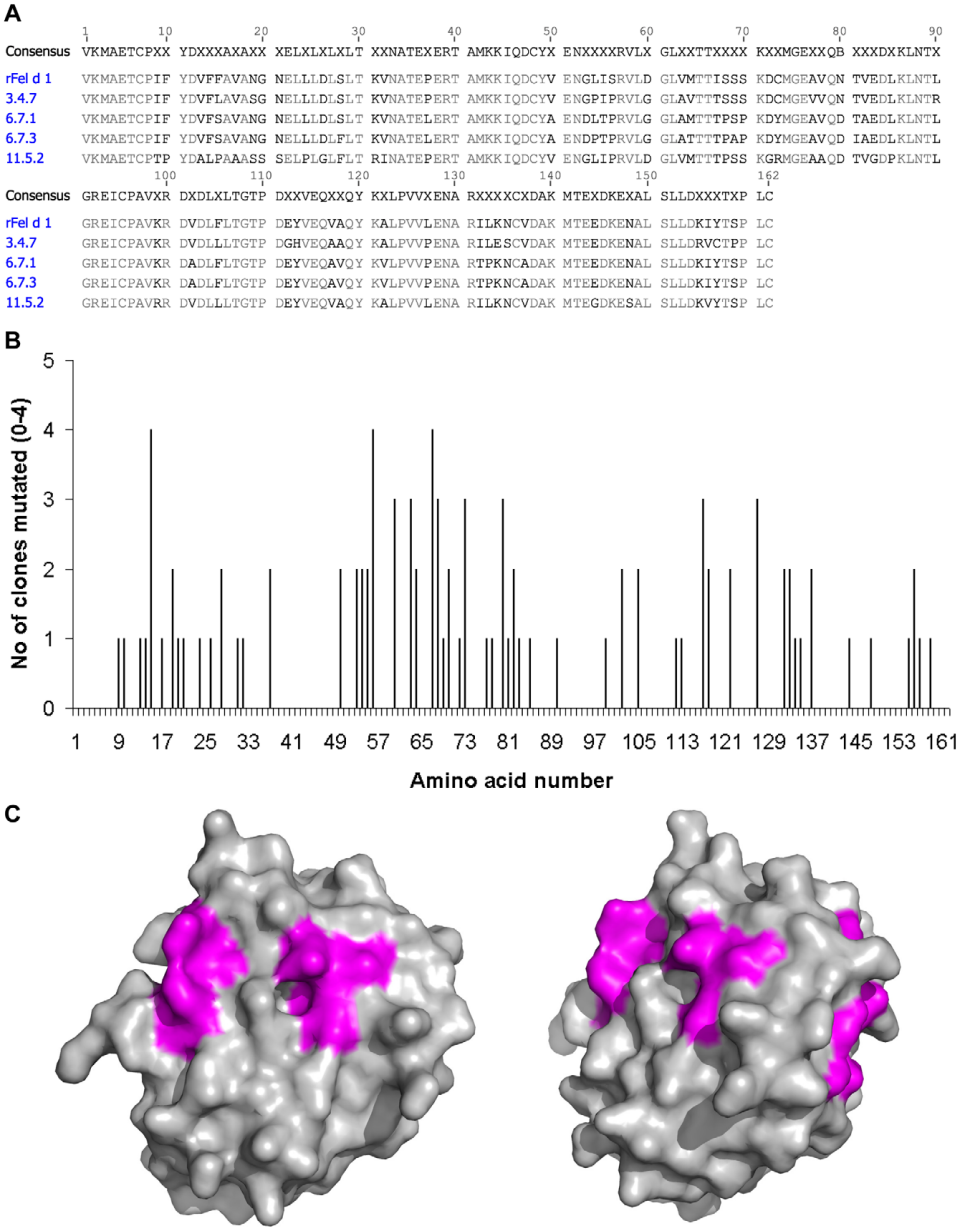
Amino acid mutation analysis. (A) Amino acid sequences of rFel d 1 in comparison to the four mutants. Deviations from the original Fel d 1 sequence are highlighted. (B) Frequency of amino acid exchanges by the four mutants compared to the original rFel d 1. Highlighted (arrows) are three areas less prone to mutations. (C) Calculated protein surface areas of Fel d 1. The three regions which are less prone to mutations are colored in magenta. The right hand side picture is rotated 90 degrees clockwise on the z-axis. Pictures were generated in Pymol (www.pymol.org).

After the first round of panning and sequencing, 3 abundant clones (clones 3, 6 and 11) were identified and used as templates for further rounds of epPCR and panning. The iterative mutational procedure for clone 3 was stopped after 4, for clone 11 after 5, and for clone 6 after 7 rounds of panning, when no or few additional mutations could be detected (Fig. 1). The final clones, denoted 3.4.7, 6.7.1, 6.7.3 and 11.5.2 contained 15 (9.3%), 19 (11.7%), 24 (14.8%) and 24 (14.8%) amino acid exchanges respectively, compared to wild type Fel d 1, (Fig. 2a). Residues 38–49, 91–98 and 138–154 appeared to be less susceptible to mutations indicating that amino acid exchanges in these areas were not randomly distributed (Fig. 2b). Further analyses of the primary structure revealed a substitution of a cysteine to a tyrosine (6.7.1, 6.7.3) or arginine (11.5.2) at residue 73, resulting in loss of a disulfide bond with Cys 95 present in rFel d 1. In addition, a bias for substitutions to residues with similar properties was noted. The most common substitution was alanine to valine or *vice versa* occurring at position 13, 17, 50, 63, 77, 78, 82, 102, 117, 118, 122 and 137 (Fig. 2a). Interestingly, certain positions seems to be particularly prone to mutate as observed for residues 28, 54, 56, 60, 63, 68, 80, 105, 117, 127 and 156 (Fig. 2b).

### Protein expression and purification

Following IPTG-induction, the four mutants and control rFel d 1 produced between, 3 and 20 mg protein per liter culture medium, and showed only small individual differences in terms of folding and aggregation. Clone 6.7.1 and 6.7.3 tended to form inclusion bodies, but were soluble after the refolding process, whereas clone 3.4.7 and 11.5.2 were produced as soluble proteins in the *E. coli* cytoplasm. Final purity of the recombinant proteins was estimated to be >95% by Coomassie-stained SDS PAGE (Fig. S1a). The LPS content of the purified proteins varied between 15 and 40 ng/mg protein.

### Protein characterization

**Figure 3:**
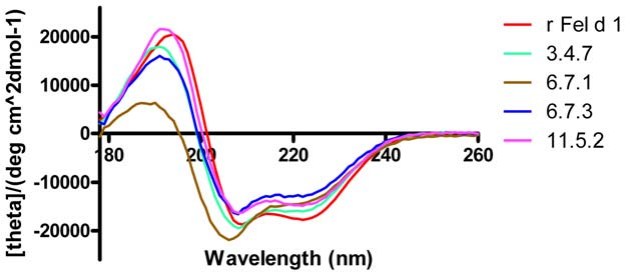
Secondary structure analysis of rFel d 1 and mutants by far-UV circular dichroism spectroscopy. The spectra are expressed as mean residue ellipticities (*θ*) at a given wavelength.

The behavior of the purified proteins in solution was analyzed by size exclusion chromatography. As reported previously for rFel d 1 [7], the proteins eluted as symmetrical peaks at approximately 30 kDa with only minor differences in elution volume (Fig. S1b) and mobility in SDS PAGE (Fig. S1a). Far-UV CD spectra recorded for rFel d 1 and the 3.4.7, 6.7.3, and 11.5.2 mutants were indicative of folded proteins with high helical content as evidenced by a double minima at 208 and 222 nm and a maximum at 190 nm (Fig. 3). The spectra of the mutants were very similar indicating an overall similar secondary structure content. Clone 6.7.1, however, displayed a less intense minimum at 222 nm and more intense and long wavelength shifted minimum at about 210 nm and a less intense maximum near 190 nm.

### IgE-binding capacity of the mutants

**Figure 4:**
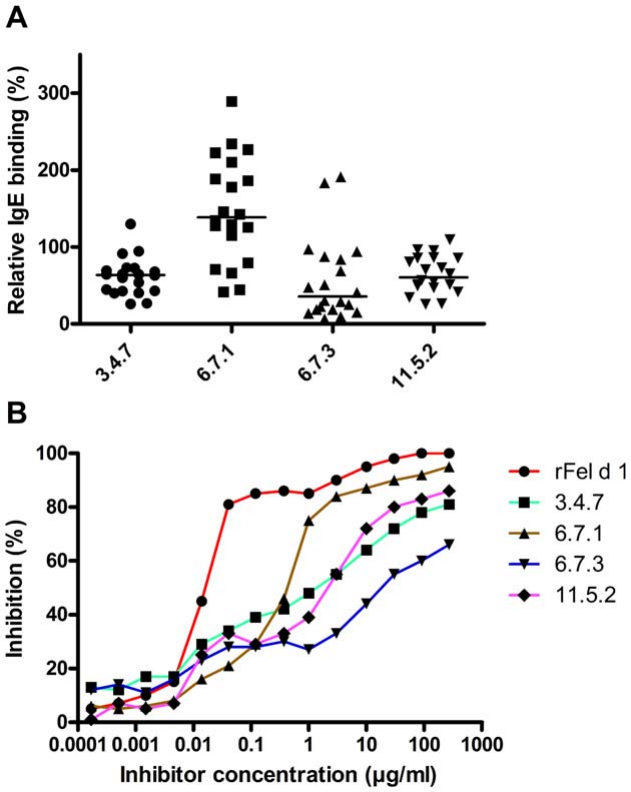
IgE binding properties of rFel d 1 and mutants by ELISA. (A) IgE responses in serum from 20 cat allergic patients to four rFel d 1 mutants were compared. The binding is shown as percentage (y-axis) of the rFel d 1 reactivity for each patient. Bars indicate medians for each group (63.5% for 3.4.7, 138.5% for 6.7.1, 35.6% for 6.7.3 and 60.7% for 11.5.2). (B). Pooled sera from rFel d 1-sensitized individuals (n = 20) were pre-incubated with three-fold dilutions (x-axis) of rFel d 1 or each corresponding mutant, followed by detection of IgE-responses. Inhibition was calculated as responses in percentage (%) after addition of antigen (y-axis).

The IgE-binding in serum from 20 cat sensitized subjects to the four mutants compared to the native-like rFel d 1 was investigated by indirect ELISA (Fig. 4a). The median IgE-reactivity and ranges in decreasing order were 6.7.1 (138.5%, range 41–289), 3.4.7 (63.5%, range 26–130), 11.5.2 (60.7%, range 26–109) and 6.7.3 (35.6%, range 7–191). In comparison to rFel d 1, clone 6.7.1 displayed the same IgE binding (P>0.05), whereas the other mutants showed significantly lower reactivity (P<0.05).

IgE-binding was also analysed by antigen competition ELISA, where a potent homologous 50% inhibition by rFel d 1 at concentration 0.5 µg/ml was obtained (Fig. 4b). The antigen concentrations needed to achieve the inhibition displayed by rFel d 1 was 30, 100, 150 and 1230 times higher for clone 6.7.1, 3.4.7, 11.5.2 and 6.7.3 respectively.

### Basophil activation test

**Figure 5:**
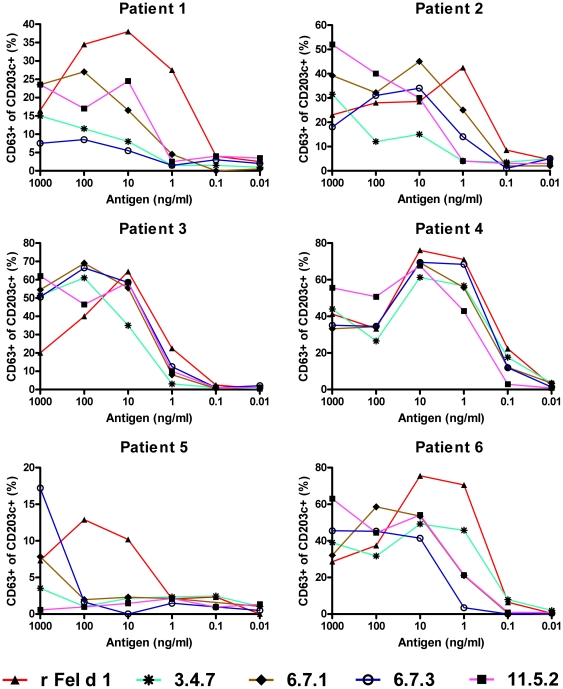
Allergenic activity analyzed by allergen stimulated basophils. Basophil activation induced by serial dilutions of rFel d 1 and mutants (x-axis) in blood from six cat allergic patients by analysis of CD63 and CD203c double positive cells (y-axis) using flow cytometry.

As a compliment to the ELISA data, allergenic IgE-reactivity of the mutants was tested *in vitro* by their capability to release mediators upon allergen cross-linking of FcεRI-bound Fel d 1-specific IgE on basophils from cat allergic patients. Analysis of the basophils by flow cytometry revealed variable reactivity and sensitivity to the allergen depending on patient and mutant tested (Fig. 5). The most potent up-regulation of CD63 was seen after stimulation with rFel d 1. The activity of protein 3.4.7 was generally low among four of the six patients tested (i.e. patients #1 (second lowest), #2 (lowest), #3 (lowest) and 5 (second lowest)), reaching a 10–1000-fold decrease compared to rFel d 1 whereas a less reduced patient-dependent degranulation activity was observed for clones 6.7.1, 6.7.3 and 11.5.2.

### T cell proliferation

**Figure 6:**
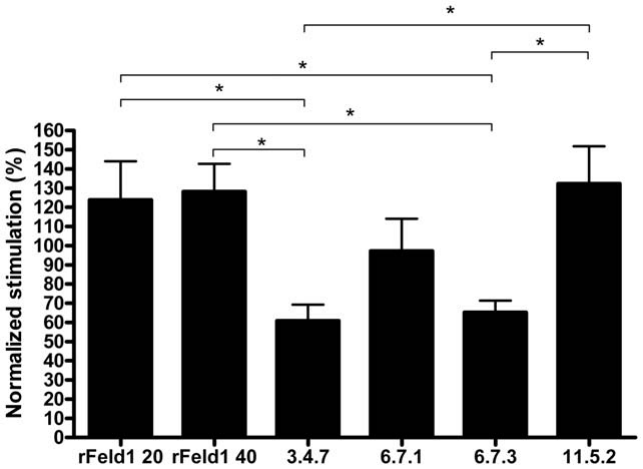
Analysis of allergen induced proliferation. Normalized proliferation (%, y-axis) measured by [^3^H] thymidine incorporation in cultured PBMC from 10 cat allergic patients after stimulation with mutants (LPS range 15–30 ng/mg protein) and two positive rFel d 1 controls containing 20 and 40 ng LPS/mg protein. Horizontal bars indicate standard error of the mean (SEM) values. * P<0.05 (Repeated measurements ANOVA with Newman-Keuls multiple comparisons test). Please note the different y-axis scale for each patient.

Cultured PBMCs from 10 cat allergic patients were stimulated with 2.5, 10 or 25 µg/ml of the mutants, influenza antigen, or rFel d 1 containing two different LPS concentrations (20 and 40 ng/mg protein). Proliferative responses were comparable between the three antigen concentrations, with the concentration 10 µg/ml showing the lowest coefficient of variation. PBMCs stimulated with mutants 3.4.7 and 6.7.3 exhibited significantly lower mean proliferation indices in comparison to PBMCs with rFel d 1, (*P*<0.05, Fig. 6). The mutant 6.7.1 showed a favorable trend towards lower induction of proliferation (P<0.2), whereas 11.5.2 showed proliferation comparable to rFel d 1. LPS levels of 20 and 40 ng/mg protein in the two rFel d 1-stimulated groups did not influence the proliferative responses. The corresponding amounts of LPS alone elicited negligible proliferation (data not shown).

### Mutants and Fel d 1-induced IgG antibody blocking capacity

**Figure 7:**
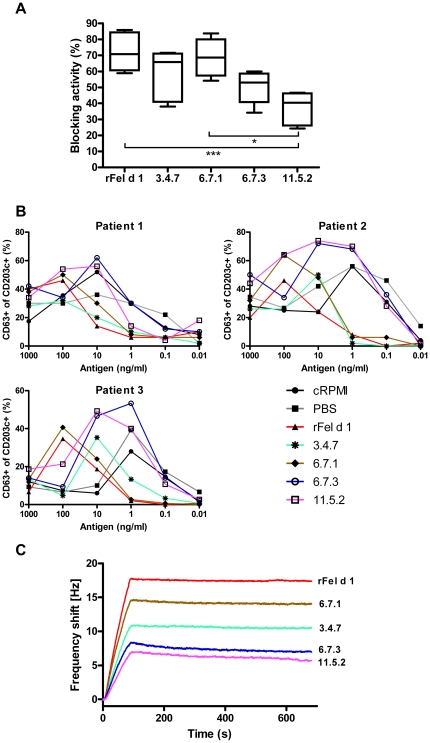
Analysis of IgG blocking antibodies induced by the four mutants and rFel d 1. (A) Pooled antiserum from mice (n = 7) immunized with respective allergen was pre-incubated in rFel d 1-coated wells, followed by addition of serum from 7 individual cat allergic patients and IgE responses recorded. The percentage of IgE inhibition is displayed on the y-axis. Boxes and horizontal bars denote 50% of values and 1 standard deviation respectively. Median values are indicated in the boxes. * P<0.05 and ***P<0.001 (Kruskal-Wallis with Dunn's multiple comparisons test). (B) Evaluation of the capacity of mutant produced IgG antibodies to inhibit rFel d 1 activation of basophils from three cat allergic patients. Serial dilutions of rFel d 1 (x-axis) were pre-incubated with titer-normalized mouse anti-mutant antisera and added to whole blood cells. Basophil activation was analyzed as percent (%) CD63 and CD 203c double positive cells (y-axis). (C) Off-rate analysis of each respective mouse antiserum pools (rFel d 1, 3.4.7, 6.7.1, 6.7.3, 11.5.2) to immobilized rFel d 1 was analysed by real-time molecular interaction using Attana. Non-specific binding of naive sera is subtracted.

Blocking antibodies of the IgG isotype have been suggested as an important mechanism behind successful SIT. Immunogenicity of rFel d 1 and each of the mutants was investigated by ELISA. Interestingly, all mutants did induce higher titers of antigen specific IgG than the wild-type protein (Fig. S2a), and importantly, all mutants except 11.5.2 induced higher titers of Fel d 1 specific IgG than rFel d 1 (Fig. S2b). The capacity of the four mutants and rFel d 1 to generate IgG antibodies that block serum IgE binding to rFel d 1 from 10 cat allergic patients was investigated by ELISA. rFel d 1 and the mutants 6.7.1 and 3.4.7 showed a similar ability to inhibit IgE-binding, whereas the blocking capacity of 11.5.2 was significantly lower than that of rFel d 1 (Fig. 7a). The ability to block serum IgE binding to rFel d 1 was also compared using basophil activation test using basophils from three cat allergic subjects. In accordance with the ELISA results, rFel d 1 and the mutants 6.7.1 and 3.4.7 showed comparable capacity to inhibit IgE-responses, while 6.7.3 and 11.5.2 showed little if any blocking effect compared to mice immunized with PBS only (Fig. 7b).

A possible explanation for the strong blocking capacity by the sera from mice immunized with 3.4.7 and 6.7.1 was revealed by ranking of all mouse sera with respect to their binding to rFel d 1. Measurement of dissociation rates showed that mice immunized with rFel d 1, 3.4.7 or 6.7.1 bound in a similar manner to rFel d 1 (kd = 7.1E^−5^, 9.1E^−5^ and 7.1E^−5^ 1/s respectively), whereas 6.7.3 and 11.5.2 showed a higher off-rate (kd = 2.6E^−4^ and 2.7E^−4^ 1/s respectively), (Fig. 7c).

**Figure 8:**
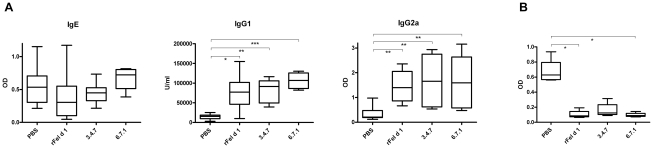
Evaluation of rFel d 1 and mutants in a mouse model for cat allergy. (A) Vaccination of rFel d 1 sensitized mice with rFel d 1 (n = 10) , 3.4.7 (n = 7), 6.7.1 (n = 8) and PBS (n = 10) in an animal model for cat allergy. IgE, IgG1 and IgG2a-antibodies measured post treatment by rFel d 1 ELISA are presented as OD values, or for IgG1 in units/ml (U/ml). (B) IgE-blocking activity of pooled sera from sensitized mice vaccinated with either rFel d 1, 3.4.7 or 6.7.1 or PBS mice on sera from PBS treated mice by IgE-blocking sandwich ELISA. IgE responses are presented as OD values. Boxes with median values and horizontal bars denote 50% of values and 1 standard deviation respectively. * P<0.05, ** P<0.01 and *** P<0.001 (Kruskal-Wallis with Dunn's multiple comparisons test).

The ability of the mutants 3.4.7 and 6.7.1 to induce an IgE blocking IgG response upon vaccination were investigated in a mouse model for cat allergy [36]. Mice were sensitized with 1 µg rFel d 1 to obtain high levels of anaphylactic antibodies, and then vaccinated s.c. with 10 µg of either 3.4.7, 6.7.1, rFel d 1 or PBS. The treatment did not increase the IgE-responses in any of the four groups and antibody responses in the non-sensitized negative control remained undetectable (not shown).. Vaccination with rFel d 1, 3.4.7 and 6.7.1 on the other hand, induced significantly higher titers of IgG1 and IgG2a than the non-vaccinated mice (Fig. 8a). Finally, the ability of pooled sera from the vaccinated groups to successfully block IgE-binding in the PBS treated group of mice (n = 5) was tested in a competitive inhibition sandwich ELISA. Unlike sera from the PBS group, the actively vaccinated groups potently blocked (rFel d 1 and 6.7.1, p-values<0.05) or showed a favourable trend (3.4.7, P-value<0.2) of blocking IgE-binding in sera from the PBS treated mice (Fig. 8b).

## Discussion

Treatments utilizing high dose hypoallergens, such as attenuated derivatives of wild type proteins or short peptides, covering the T cell repertoires of allergens have been tested in clinical trials. However, such strategies have induced LPRs, where symptoms evolve several hours after administration.

The aim of this study was to develop a technology for the *in vitro* evolution of allergens with a native-like structure for a maintained immunogenicity and treatment efficacy, but with reduced IgE-binding and T cell activation for reduction of immediate and late phase side effects during allergen-specific immunotherapy. Numerous studies have demonstrated sensitization to allergens as a risk factor for the development of allergy and asthma. In more severe cases, asthma is a chronic condition without curative therapeutic options. An alternative rarely considered for this group of patients is alleviation of allergic and asthmatic symptoms by SIT. One of the reasons for the restricted use of SIT in the treatment of extrinsic allergic asthma is the risk of adverse reactions which even may have a fatal outcome [37].

Novel candidate vaccines would be expected to have a high safety profile including reduced immediate side effects due to a decrease in IgE-mediated degranulation of effector cells and a reduced capability of the TCE to activate T cells, which could result in mild or absent LPR. We hypothesized that the conservation of a native like tertiary structure with concomitant reduction of the IgE-binding capacity would keep the ability to induce strong protective allergen-specific IgG immune responses, which in turn would be able to compete with IgE for epitope binding on the native allergen. The use of freshly prepared serum pools were used to reduce the risk of epitope biased selection.

We used Fel d 1, the major and most dominant cat allergen, as a prototype allergen to test the feasibility of our approach. In a first step the sequence coding for rFel d 1 [7] was submitted to epPCR amplification to construct a complex phage surface displayed library of mutated clones. Phagemids were isolated by an iterative epPCR and biopanning procedure over solid-phase immobilized IgE from selected patients.

Four candidate sequences derived from three different panning experiments showing the highest numbers of mutations compared to the rFel d 1 sequence (Fig. 2) were chosen for detailed investigation. All mutant proteins were well expressed by *E. coli* either as inclusion body (clones 6.7.1 and 6.7.3) or as soluble cytoplasmic proteins (clone 3.4.7 and 11.5.2), folded well using established methods [7] and eluted as symmetrical peaks around 30 kDa by size exclusion chromatography as observed for Fel d 1. Circular dichroism analysis of the mutants showed spectra comparable to those of rFel d 1 except for clone 6.7.1 which displayed a less intensive maximum at 190 nm (Fig. 3). The similarity of rFel d 1 and mutant spectra, with the possible exception of clone 6.7.1 is evidence, albeit low resolution, that the mutants are indeed folded similarily as rFel d 1 allowing structural inferences to be made from sequence data.

The IgE-binding capacity of the mutants 3.4.7, 6.7.3 and 11.5.2 was significantly reduced compared to rFel d 1, while the IgE-reactivity of 6.7.1 was not changed (Fig. 4a), as evaluated by ELISA. Similar results were also observed when analyzing the capacity of the mutants to inhibit IgE from a pool to complex with solid phase bound rFel d 1 by ELISA (Fig. 4b). However here we received supporting *in vitro* data from the basophil activation assay. The mutant 6.7.1 generally displayed the highest IgE-binding and degranulating capacity, while the other mutants showed a weaker binding to IgE, with 3.4.7 generally showing the lowest degranulating capacity. We assume that the high number of mutations introduced has reduced the number or conformation of IgE epitopes of Fel d 1 by substitution of amino acids essential for interaction with IgE. We are, however, not able to confirm this hypothesis because no convincing information about conformational IgE epitopes on Fel d 1 is available. Certainly, due to the selection procedure based on serum IgE from cat allergic patients, at least one IgE-binding epitope has to be retained by all mutants. A reduction of the number of IgE-binding epitopes can explain the reduced IgE-binding activity of the mutants and in agreement, all mutants showed a reduction in the capacity to stimulate activation of basophil from cat allergic patients demonstrating the hypoallergenic potential of these mutants (Fig. 5). Mutants 3.4.7 and 6.7.3 generally showed the highest reduction of activation regarding the amount of allergen needed to induce degranulation (sensitivity) and the percentage of basophils degranulating (reactivity). Interestingly, this pattern was more clearly noted in patients with lower basophil reactivity. All mutants showed a hypoallergen pattern by basophil activation but showed a varied pattern between patients, which may call for caution when choosing the most optimal mutant if used for treatment. In further experiments we tested if the mutational procedure is able to generate proteins with reduced activation of allergen-specific T cells. Therefore, we measured the T cell proliferation of the vaccine candidates in PBMC cultures from cat allergic patients. Again, clones 3.4.7 and 6.7.3 showed significantly reduced T cell activation profiles whereas clones 6.7.1 and 11.5.2 showed a T cell activation capacity comparable to those of rFel d 1, despite up to 15% mutated amino acids (Fig. 6).

Notably, successful immunotherapy is paralleled by an increased production of allergen-specific IgG antibody titers and, therefore, we tested the ability of the mutants to induce potent IgE-blocking antibodies in mouse immunization experiments. As clearly shown, all mutants were able to induce IgG-anti Fel d 1 IgE blocking antibodies in immunized mice, confirming a widely conserved immunogenicity and antigenicity of the variants 3.4.7 and 6.7.1, thus indicating their potential as vaccine candidates (Fig. 7, Fig. S2). The mutants' capacity to induce blocking IgG antibodies is illustrated by the real-time binding data (Fig. 7c). Using comparable titers, the total shift (Hz) differed between the wild type and each of the mutants, with rFel d 1 showing the highest binding, followed by 6.7.1, 3.4.7, 6.7.3 and 11.5.2. Thus, a greater number of IgG-antibodies induced by the wild type protein also bind to the wild type protein compared to antibodies induced by the mutants. Does the reduced epitope recognition translate into reduced effectiveness of the mutagenic proteins in terms of blocking capacity? Importantly, both the IgG blocking of human IgE by ELISA (Fig. 7a) the basophil degranulation test (Fig. 7b) and the mouse IgE-blocking sandwich ELISA after vaccination (Fig. 8b) show mutants 3.4.7 and 6.7.1 to be as effective as rFel d 1 in terms of blocking capacity. In addition, the mouse anti cat allergy model displayed a similar antibody profile of IgE, IgG1 and IgG2a antibodies after vaccination with 3.4.7 and 6.7.1, in a similar manner as that of the wt rFel d 1 (Fig. 8a). The results suggest that the total amount of specific antibodies bound to an antigen does not necessarily relate to a beneficial biologic response. The off-rates are similar between rFel d 1, 3.4.7 and 6.7.1, while 6.7.3 and 11.5.2 dissociate more rapidly from rFel d 1 (Fig. 7c). This indicates a strong cross-reactivity, similar to that of rFel d 1, by antibodies from 3.4.7 and 6.7.1.

Interestingly, in comparison to the wild type molecule, the real-time Attana data suggests that the epitope repertoire produced by the mutants 3.4.7 and 6.7.1 the still cover all the major IgE-binding epitopes Moreover, with the exception of 11.5.2., the mutants were highly immunogenic. in that they induced higher titres of rFel d 1-specific IgG-antibodies than the wild type molecule (Fig. S2b) which would be a desirable a feature by SIT.

The advantage of combining random mutations and phage display is that unbiased selection of desirable molecular properties. Detailed knowledge as structure, stability, productivity and of the complex interactions between individual amino acids in a given protein and of the structure-function relationships of the target molecules is of less importance.

In summary, by consecutive rounds of error prone PCR followed by selection from phage displayed libraries we have isolated folded Fel d 1 mutants with reduced IgE-binding and T cell activation capacity, as safer alternatives for use in SIT. These allergens retained the ability to induce strong IgG-mediated protective responses by mouse immunization experiments as shown in particular for mutants 3.4.7.and 6.7.1. We conclude T and B cell epitope hypoallergens thus evolved resemble many desirable features of promising vaccine candidates for safer and efficacious treatment of cat associated allergy and asthma.

## Materials and Methods

### Patients and control subjects

Randomly chosen sera from 20 patients with positive serum IgE-responses to cat dander by ImmunoCAP (Phadia, Uppsala, Sweden), were used to analyze IgE-binding to the Fel d 1 mutants and to rFel d 1 used as positive control. A pool from these sera was prepared for use in allergen competition ELISA.

New serum pool from 3–4 cat-sensitized subjects were mixed for each round of biopanning. The inclusion criterion was a cat dander specific IgE content exceeding 10% of the total IgE as measured by ImmunoCAP (Phadia). Serum pools from healthy individuals without serum IgE to cat dander were used as negative controls. Sera mentioned above were collected at the routine allergy diagnostic lab at Karolinska University hospital. Ten consecutively selected well-characterized cat allergic and asthmatic patients were recruited at the Karolinska University Hospital, Lung Allergy clinic, and PBMC were isolated [38] for determination of allergen-specific proliferation. Venous blood was collected from six cat allergic patients for basophil activation test, and blood from another three cat allergic patients was obtained to analyse the inhibitory effect on degranulation of mouse IgG anti-mutant antibodies on rFel d 1 activated basophils.

The study was approved by the local ethics committee and all patients gave an informed written consent.

### ELISA and inhibition ELISA

Solid-phase ELISA measurements were carried out essentially as described [7]. Briefly, antigen or antibodies was coated to microtiter wells (Nunc, Roskilde, Denmark). Sera from patients, IgE-myeloma containing 2000 kU/L IgE serving as negative control and phages were incubated in serial dilution followed by detection of bound IgE-antibodies [39]. A reference curve was included in each run for quantification of IgE [40].

Allergen inhibition ELISA was performed as described above, but with a prior over night incubation at +4°C of serum from a pool of cat sensitized patients (n = 20) mixed with 3-fold serial dilutions (range 170 pg/ml–270 µg/ml) of mutants or rFel d 1 as competing antigen.

### Phage display libraries

Libraries containing random mutations were created by epPCR using dNTP-Mutagenesis Kit (Jena Bioscience, Jena, Germany). The rFel d 1 [7] template used in the first round was amplified with the rFel d 1-specific primers 5′ caccggaatt cgttaaaatg gctgaaacct gc and 5′ cgcgccgctc gaggcacagc gggg, with overhangs designed for the Eco RI/Xho I cloning sites of phagemid pJuFo [41]. The purified PCR amplicons were inserted into the rescue vector pT7-Blue (Novagen Inc., Madison, WI, USA), using pT7 Blue Perfectly Blunt Cloning Kit (Novagen), transformed into Nova Blue Singles cells (Novagen), vectors isolated (Plasmid Miniprep Spin Kit, Genomed, Löhne, Germany) and fragments excised using Eco RI and Xho I restriction sites, subsequently ligated into pJuFo using T4 ligase (New England Biolabs, Ipswich, MA, USA).

Phagemids carrying the mutated Fel d 1 coding sequences were transformed into XL1-Blue MRF' Electroporation-Competent Cells (Stratagene, La Jolla, CA, USA) by electroporation (Bio-Rad, Herts, United Kingdom), immediately added to 3 ml SOC medium for 1 h at 37°C followed by titration. Transformed cells were added to 12 ml 2×YT medium containing ampicillin (100 mg/L) and tetracycline (10 mg/L) and incubated for 2 h (250 rpm, at 37°C) before addition of helper phage (VCSM13, Stratagene) at a multiplicity of infection (moi) of 10. The medium was supplemented with kanamycin (25 mg/L) after an additional 2 h and the culture incubated over night. The steps described above were repeated 4–7 times depending on the clone.

### Selection of clones

Phage-infected XL1-Blue cell were centrifuged (J2-21 Beckman Coulter, Palo Alto, CA) at 5000× g, 15 min, 4°C. The supernatant was collected and phagemids precipitated with 5% PEG-8000, 4% NaCl final concentration, incubated on ice for 2 h followed by centrifugation (18600× g, 15 min, 4°C). Phages were resuspended in ice cold Tris-buffered saline (TBS) centrifuged (16100× g, 10 min, 4°C) to eliminate cell debris and supernatants stored at 4°C.

100 µl, 2 µg/ml of monoclonal anti-human IgE (a kind gift from Phadia, Uppsala, Sweden) was coated to 96-well microtiter plates (Nunc). 100 µl of a patient serum pool (n = 3) containing Fel d 1-specific IgE, Fel d 1 negative serum, or TBS as negative control was added with shaking at 100 rpm for 2 h at 37°C. After washing, phage library (100 µl, around 10^5^ cfu) was added and incubated with shaking at 100 rpm for 2 h at 37°C. The supernatant containing unbound phages was discarded and the wells washed using TBS with 0,1% Tween with increasing stringency (5, 10 and 15 times), to isolate clones with the highest IgE- binding capacity. Bound phages were eluted with 0.1 M glycin buffer, pH 2.2, neutralized with 0.5 M Tris-HCl pH 10.9, and added to 5 ml of exponentially growing XL1-Blue, optical density (OD) 1.0 at 600 nm. Number of phages before (input) and after biopanning (output) were calculated by titrations counting single colony forming units on agar plates. To prepare phage for the next round of panning ampicillin (100 mg/L) and tetracycline (10 mg/L) were added to the infected XL1-Blue cells. Helper phage (moi of 10) and kanamycin (25 mg/L) were added after 2 h and the cultures incubated over night at 37°C with shaking at 250 rpm.

### Sequencing

The inserts of twelve randomly picked colonies from the input and output of each panning round were amplified by colony PCR with pJuFo sequencing primers 5′ ccgaaatcgc gaacctgc and 5′ aacgacggcc agtgaattg. The PCR products were purified (PCR Product Purification Spin Kit/250, Genomed) and used as templates for sequencing (BigDye Terminator v1.1 Cycle Sequencing Kit and ABI PRISM 310 Genetic Analyzer, Applied Biosystems, CA, USA). The repetitive biopanning process was terminated when no new dominant clones could be amplified, as confirmed by sequencing of 12 randomly picked clones.

### Expression of recombinant allergens

Finally selected clones were PCR-amplified using rFel d 1 primers, cleaved by XhoI and NdeI restriction endonucleases and ligated into the expression vector pET20b (Novagen). The constructs were transformed into BL21(DE3)pLysS (Novagen), plated on LB plates containing 100 mg/L ampicillin and 35 mg/L chloramphenicol and incubated overnight at 37°C. Single colonies were picked, cultured in 15 ml LB medium containing ampicillin (100 mg/L) and chloramphenicol (35 mg/L) overnight at 37°C and 250 rpm and used to inoculate two liters of LB medium containing ampicillin (100 mg/L) and chloramphenicol (35 mg/L) for protein production. Cells at log phase were induced using isopropyl thiogalactoside at a final concentration of 0.4 mM, with simultaneous addition of ampicillin to 100 mg/l final concentration. After 3 h incubation at 37°C, the cells were collected by centrifugation (18600× g, 10 min), resuspended in 30 ml PBS and frozen at −20°C.

### Protein purification

Thawed pellets were adjusted to 6 M guanidine-HCl, sonicated (Soniprep 150 ultrasonic disintegrator, Sanyo Gallenkamp, Uxbridge, UK) by 5 bursts×10 sec (10 ma) on ice and centrifuged at 18600× g for 10 min. Buffer was exchanged on Sephadex G-25 equilibrated in 5 mM Tris-HCl containing 0.5 M NaCl, 20 mM imidazol and 6 M guanidine-HCl, loaded on a 5-ml Ni^2+^-HiTrap IMAC column (GE Healthcare, Uppsala, Sweden) followed by elution with 5 mM Tris-HCl containing 0.5 M NaCl, 0.5 M imidazol and 6 M guanidine-HCl. The guanidine was removed by dialysis in 20 mM Tris-HCl, pH 7.4. Proteins were concentrated to 3 ml in a 10 ml Amicon cell (Millipore, Billerica, MA, USA) prior to size exclusion chromatography on a 16/60 Superdex 200 pg column equilibrated in PBS pH 7.4 using FPLC system (GE Healthcare). The peaks corresponding to monomeric proteins (30 kDa) were diluted 1∶1 in 20 mM Tris-HCl pH 8.7, and loaded on 10 ml anion exchange Q-Sepharose HP columns (GE Healthcare) equilibrated in 20 mM Tris-HCl pH 8.7. Proteins were fractioned with a 15 column volumes linear gradient using 20 mM Tris-HCl, 1 M NaCl, pH 7.4, aliquoted and stored at −80°C. Protein concentration was determined using the BCA protein assay (Thermo scientific, Chicago, USA) using bovine serum albumin as standard. The purity was estimated by SDS-PAGE under reducing and non-reducing conditions, and by analytical SEC using a Superdex 200 32/300 column (GE Healthcare) equilibrated in PBS at a flow rate of 40 µl/min using ÄKTA Purifier (GE Healthcare).

For control of allergen-specific T cell proliferation, two samples of purified rFel d 1 containing 20 and 40 ng lipopolysaccharide (LPS)/mg protein, respectively, were used. The LPS content was determined with the limulus amebocyte lysate endochrome assay according to the instructions (Charles River Endosafe, Charleston, USA).

### Protein characterization

SDS-PAGE was performed under reducing conditions as described [7]. Analytical SEC was performed using ÄKTA Purifier (GE Healthcare) and a Superdex 200 PC 3.2/30 column, equilibrated in PBS pH 7.4. The secondary structure of the purified mutants and rFel d 1 was analyzed by far-UV circular dichroism (CD) spectra measured at 25°C in phosphate buffer (10 mM NaHPO4, pH 7.4). Data were collected at 1 nm intervals with time averaging for 16 seconds using a bandwidth of 1.5 nm in a 0.01 cm quartz cuvette (Hellma, Müllheim, Germany) using an Aviv 202 DS spectropolarimeter (Aviv Biomedical, Lakewood NJ, USA).

### Basophil activation test (BAT)

Allergen-specific basophil degranulation was analyzed by monitoring the basophil activation markers CD203c and CD63 using flow cytometry [42]. Briefly, 10-fold serial dilutions of antigen (1000 ng/ml to 0.01 ng/ml), medium (negative control) and anti-IgE (positive control) was added to venous blood samples from three cat allergic patients and incubated with anti-CD63 and anti-CD203c (Immunotech, Marseille, France). From each patient, allergen-activated basophils were identified by gating for CD203c^+^ cells using FACS Calibur (BD Biosciences, San Jose, CA, USA). The magnitude of reactivity (y-axis, Fig. 5) was reported as percentage of CD63^+^ cells among the gated basophils. Sensitivity was analyzed at the right hand side of the bell-shaped BAT curve, where positive reactivity was measured post stimulation with the wild type protein. Comparison of the rFel d 1 mutants with the wild type protein was analyzed at 50% of reactivity with rFel d 1 within the sensitive range.

### T cell proliferation

Allergen-specific proliferation of cultured PBMC from 10 cat allergic patients was analyzed in triplicates with 2×10^5^ cells/well using 96-well plates [38]. Cells were stimulated with either 2.5, 10 or 25 µg/ml of the Fel d 1 mutants and rFel d 1 containing 20 or 40 ng LPS/mg protein and influenza antigen, (Vaxigrip® vaccine, Aventis Pasteur, Lyon, France) 30 ng/ml LPS in cRPMI and cRPMI alone as controls. Cells were incubated in 5% CO_2_ for five days at 37°C when 1 µCi/well [^3^H] thymidine was added followed by 18 h of incubation. Counts per minute (cpm) obtained from the cell cultures were divided by cpm values from unstimulated wells and expressed as a stimulation index (SI). SI values ≥2.0 were considered to be positive. The proliferation varied between patients (SI range, 5–80) and was normalized for purposes of comparison. For each patient, a mean proliferation value (MPV, cpm for tested antigens/number of wells) was calculated. This value was set to 100%. The stimulation for single antigens is presented as percentages in relation to the MPV.

### Assessment of IgE-blocking mutant induced IgG antibodies

Six to eight week old female BALB/c mice were obtained from Charles River (Sulzfeld, Germany) and housed with food and water *ad libitum*. Animal experiments were approved by the Swedish local ethics committee for animal welfare.

The ability of the four mutants to generate IgE-blocking IgG antibodies was tested. Groups of mice (n = 4) were immunized subcutaneously in the neck with 10 µg of the mutant proteins and as positive control rFel d 1, each protein adsorbed to 1 mg aluminium hydroxide (Sigma-Aldrich, Steinheim, Germany). Injections were given on day 0, 14, 28 and 42. On day 47, mice were sacrificed and blood collected. Sera from mice immunized with a distinct protein were pooled and a serum pool from naïve mice was used as a negative control.

In a first set of experiments titers were normalized between the five different mouse immune serum pools by 5-fold serial dilutions from 1/50 to 1/781250 using the respective antigen by ELISA as described (Fig. S2a) [7].

The ability of mouse IgG-antibodies to block binding of patient's serum IgE to allergen was determined by ELISA. Plates were coated with rFel d 1 and blocked with BSA. The normalized mouse serum pools were added and incubated over night at 4°C. The following day, patient serum containing IgE to rFel d 1 diluted to 0.85 kU_A_/L, and serum from an IgE-myeloma of 2000 kU/L as negative control was added, followed by IgE detection as described.

To assess the biological relevance of anti- mutant IgG, the ability to inhibit Fel d 1-mediated degranulation of basophils was investigated. Briefly, 10-fold serial dilutions of rFel d 1 (1000 ng/ml to 0.01 ng/ml) or medium was pre-incubated over night with the four normalized mouse anti-mutant IgG serum pools and the rFel d 1 pool as control. The mixes of antigen and serum pool were then added to fresh venous blood from three cat allergic patients as described above.

For real-time analysis of the different mouse antisera with respect to binding properties to rFel d 1 an Attana A200 system (Attana AB, Sweden) was used. rFel d 1 (5 µg/mL in 10 mM sodium actetate pH 4.5) was immobilized on a LNB sensor chip by amine coupling according to the manufacturer's instructions. All experiments were performed in running buffer, HBS-T (10 mM HEPES buffered saline with 0.005% Tween 20), at 37°C. Dissociation rate constants for the 5 different antisera were determined at 25 µL/min using the Attester Evaluation off-rate screening tool, between time points 150 and 450 seconds. All sera were normalized as determined by ELISA, and diluted 1∶100 in HBS-T. The surface was regenerated using a 41 s pulse of 10 mM glycine pH 1.5. Non-specific binding to the activated and deactivated LNB carboxyl surface was recorded using sera from mice immunized with PBS.

### Vaccination using mutants in a mouse model for cat allergy

Animals were obtained and housed as described above. Mice were sensitized s.c. with 1 µg, rFel d 1 adsorbed to 1 mg alum in 200 µl PBS or PBS only (non-sensitized negative control) on day 0, 14 and 28 to obtain high titers of anaphylactic antibodies. Groups of mice (n = 7–10) were then vaccinated s.c. with 10 µg of rFel d 1, 3.4.7) or 6.7.1 respectively in 200 µl PBS or 200 µl PBS alone (control) on day 30, 32 and 34. The mice were sacrificed on day 42 and peripheral blood was collected and stored at −80°C

Levels of Fel d 1-specific IgE, IgG1 and IgG2a was analyzed by ELISA. For analysis of IgE, 96-well plates (Nunc) were coated with 0.2 µg/100 µl rat anti-mouse IgE (BD Biosciences), and IgG1 and IgG2a plates were coated with 0.5 µg rFel d 1. Sera were diluted 1∶20 for IgE measurement, 1∶25000, 1∶100000 or 1∶1000000 for IgG1 and 1∶1000 for IgG2a. Levels of IgG 1 anti rFel d 1 was analysed using an in-house reference curve and presented in units/ml, U/ml. Bound IgE levels was measured using 0.2 µg/100 µl and well of biotinylated rFel d 1 followed by alkaline phosphatase (AP)-conjugated streptavidine (diluted 1∶3000, Sigma-Aldrich). IgG1 and IgG2a levels were detected using goat anti-mouse AP-conjugated IgG1 (1∶5000) and IgG2a (1∶1000) respectively (Southern Biotech, Birmingham, AL, USA), followed by OD measurements at 405 nm.

The IgE blocking capability of the antibodies obtained after treatment was investigated using a competitive inhibition sandwich ELISA as described above. Pooled sera from the group of mice treated with PBS only were selected and added to wells coated with rat anti-mouse IgE. Fresh serum pools from the respective groups were prepared, diluted 1∶10 and pre-incubated over night with biotinylated rFel d 1 protein to a final concentration of 2 µg/ml. The pre-incubated protein mixes were then added to wells and detection of bound biotinylated rFel d 1 was carried out.

### Statistical analysis

Proliferation data was analysed by ANOVA with Newman-Keuls multiple comparisons test for pair wise analysis and the ELISA and mouse study analysed by Kruskal-Wallis with Dunn's multiple comparisons test. All statistics were performed using Graphpad Prism 5.02 software (Graphpad Software Inc., San Diego, CA, USA). P-values<0.05 were considered significant.

### Ethics Statement

All animal research has been approved by the local ethics committee at Karolinska University Hospital, “Stockholms norra djurförsöksetiska nämnd”, with the approval ID [DNR: N245/08]. All human research has been approved by the local ethics committee at Karolinska Institutet, “Regionala etikprövningsnämnden i Stockholm”. Written consent was obtained from all participants involved in the present study.

## Supporting Information

Figure S1 **Size and purity of rFel d 1 and mutants.** (A) SDS-PAGE of purified rFel d 1 mutants under reducing conditions with comassie blue staining. Molecular weight markers (lane 1,7), rFel d 1 (lane 2), 3.4.7 (lane 3), 6.7.1 (lane 4), 6.7.3 (lane 5), 11.5.2 (lane 6). (B) Analytical size exclusion chromatography of rFel d 1 and mutants. The dots denote bovine serum albumin (*67 kDa*), ovalbumin (*43 kDa*), and chymotrypsinogen A (*25 kDa*) used as molecular weight markers.
(TIF)

Figure S2 **Comparison of immunogenicity of rFel d 1 and mutants.** Serial dilutions (x-axis) of pooled mouse sera from mice immunized with either rFel d 1 or each respective mutant. Pre-diluted sera were added to 96-well microtiter plates coated with either (a) respective allergen or rFel d 1 only (b), and IgG responses (y-axis) measured by ELISA.
(TIF)
