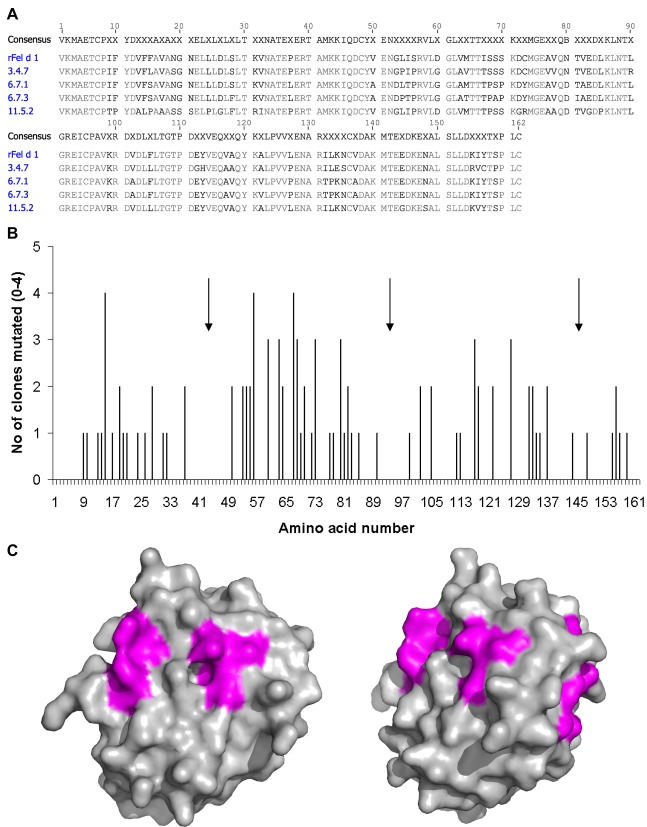# Correction: In Vitro Evolution of Allergy Vaccine Candidates, with Maintained Structure, but Reduced B Cell and T Cell Activation Capacity

**DOI:** 10.1371/annotation/faa4bade-b4a4-422e-9554-57908a0ae516

**Published:** 2012-05-08

**Authors:** Ola B. Nilsson, Justus Adedoyin, Claudio Rhyner, Theresa Neimert-Andersson, Jeanette Grundström, Kurt D. Berndt, Reto Crameri, Hans Grönlund

There was an error in Figure 2. The arrows were missing from panel B. The correct Figure 2 can be viewed here: 

**Figure:**