# Supplementary figures and images for: In Vitro Evolution of Allergy Vaccine Candidates, with Maintained Structure, but Reduced B Cell and T Cell Activation Capacity
Source: PLoS One. 2011 Sep 13;6(9):e24558. doi: 10.1371/journal.pone.0024558 (PMC3172221; doi:10.1371/journal.pone.0024558)

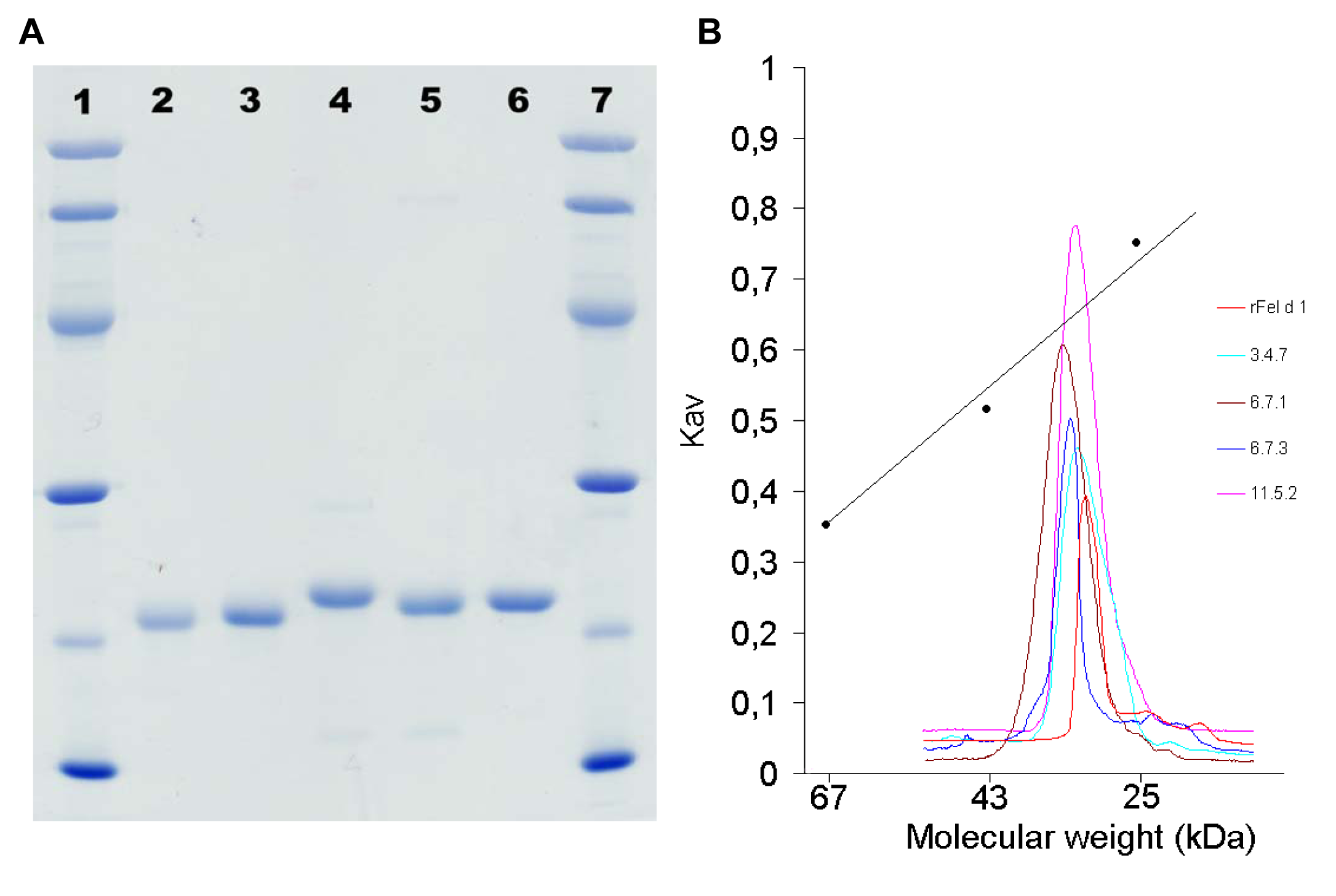

Supplement: Figure S1 — Size and purity of rFel d 1 and mutants. (A) SDS-PAGE of purified rFel d 1 mutants under reducing conditions with comassie blue staining. Molecular weight markers (lane 1,7), rFel d 1 (lane 2), 3.4.7 (lane 3), 6.7.1 (lane 4), 6.7.3 (lane 5), 11.5.2 (lane 6). (B) Analytical size exclusion chromatography of rFel d 1 and mutants. The dots denote bovine serum albumin (67 kDa), ovalbumin (43 kDa), and chymotrypsinogen A (25 kDa) used as molecular weight markers. (TIF) [file pone.0024558.s001.tif]
